# CRISPR/Cas9-mediated mutagenesis of homologous genes in Chinese kale

**DOI:** 10.1038/s41598-018-34884-9

**Published:** 2018-11-14

**Authors:** Bo Sun, Aihong Zheng, Min Jiang, Shengling Xue, Qiao Yuan, Leiyu Jiang, Qing Chen, Mengyao Li, Yan Wang, Yong Zhang, Ya Luo, Xiaorong Wang, Fen Zhang, Haoru Tang

**Affiliations:** 1 College of Horticulture, Sichuan Agricultural University, Chengdu, 611130 China; 2 Institute of Pomology and Olericulture, Sichuan Agricultural University, Chengdu, 611130 China

## Abstract

The clustered regulatory interspaced short palindromic repeat-associated protein 9 (CRISPR/Cas9) system has developed into a powerful gene-editing tool that has been successfully applied to various plant species. However, studies on the application of the CRISPR/Cas9 system to cultivated *Brassica* vegetables are limited. Here, we reported CRISPR/Cas9-mediated genome editing in Chinese kale (*Brassica oleracea* var. *alboglabra*) for the first time. A stretch of homologous genes, namely *BaPDS1* and *BaPDS2*, was selected as the target site. Several stable transgenic lines with different types of mutations were generated via *Agrobacterium*-mediated transformation, including *BaPDS1* and *BaPDS2* double mutations and *BaPDS1* or *BaPDS2* single mutations. The overall mutation rate reached 76.47%, and these mutations involved nucleotide changes of fewer than 10 bp. The clear albino phenotype was observed in all of the mutants, including one that harbored a mutation within an intron region, thereby indicating the importance of the intron. Cleavage in Chinese kale using CRISPR/Cas9 was biased towards AT-rich sequences. Furthermore, no off-target events were observed. Functional differences between *BaPDS1* and *BaPDS2* were also assessed in terms of the phenotypes of the respective mutants. In combination, these findings showed that CRISPR/Cas9-mediated targeted mutagenesis can simultaneously and efficiently modify homologous gene copies of Chinese kale and provide a convenient approach for studying gene function and improving the yield and quality of cultivated *Brassica* vegetables.

## Introduction

Genome-editing techniques using sequence-specific nucleases (SSNs) provide exceptionally powerful tools that cause targeted gene knockout or knockdown by introducing base insertions, deletions, or substitutions into specific DNA sequences, which enable precise genomic sequence modification. To date, several genome editing techniques have been reported, such as zinc finger nucleases (ZFNs)^1–3^, transcription activator-like effector nucleases (TALENs)^4,5^, and the more recent clustered regulatory interspaced short palindromic repeat (CRISPR)-associated protein 9 (CRISPR/Cas9)-based methods^6–8^. ZFNs are a fusion of the transcription factor protein ZFA (Zinc finger activator) and FOKI (a endonuclease isolated from the bacterium *Flavobacterium okeanokoites*), while TALENs are constructed by fusing transcription activator-like (TAL) effectors and FOKI^9^. ZFNs and TALENs both can specifically recognize and bind to the specified DNA sequence to form a cleavable FOKI dimer, and then induce DNA double-strand breaks (DSBs) at the target site^4^. ZFNs and TALENs have been successfully applied in editing plant genomes, but there still exist some deficiencies like consuming time, expensiveness and high complexity of vector construction^10^. CRISPR/Cas9 derived from an adaptive immune system in bacteria and archaea, and it can perform precise genome editing of target genes in a variety of organisms through artificial manipulation. Due to its low cost, design flexibility, versatility, and high efficiency, the CRISPR/Cas9 system provides a solution for the drawbacks of ZFNs and TALENs, and has become the third-generation genome editing technique following ZFNs and TALENs^11^.

The principle of CRISPR/Cas9 is that CRISPR RNA (crRNA) binds to *trans* activating CRISPR RNA (tracrRNA) by base pairing to form a tracrRNA/crRNA binary complex, which guides the endonuclease Cas9 to cleave the target double-stranded DNA. The tracrRNA/crRNA binary complex can be substituted by an engineered single guide RNA (sgRNA)^12^. Critically, the Cas9 protein recognizes an NGG protospacer adjacent motif (PAM) which flanks the 3′ end of the target sequence, and then achieve the cleavage of the target site^13^. The resulting DSBs trigger non-homologous end joining (NHEJ) or homologous recombination (HR) repair pathways, which may lead to gene mutations^14^. If an insertion, deletion, or substitution occurs at the coding region of the gene, it will probably cause a frameshift mutation in the target gene and may remarkably affect gene function^15^.

CRISPR/Cas9 technology has been widely and successfully applied to host DNA mutagenesis in a variety of plants, such as *Nicotiana benthamiana*^16^, *Arabidopsis*^17^, wheat^18^, rice^11^, Zea^19^, sorghum^20^, potato^21^, sweet orange^22^, tomato^23^, grape^24^, populus^25^, citrus^26^, and apple^27^. However, despite the existence of a diverse range of *Brassica* plants, the CRISPR/Cas9 system has only been successfully applied to oilseed rape^28,29^ and *Brassica oleracea*^30^. *Brassica* vegetables are a large group of agriculturally important vegetables that have high nutritional and economic value. Chinese kale (*B. oleracea* var. *alboglabra*) is an original Chinese *Brassica* vegetable that is widely distributed in southern China and Southeast Asia. In addition to its good flavor, Chinese kale also has high nutritional value due to its high levels of antioxidants and anticarcinogenic compounds, including vitamin C, carotenoids, total phenolics, and glucosinolates^31,32^. Here, we report the application of the CRISPR/Cas9 technology in Chinese kale (a cultivated *Brassica* vegetable) for the first time.

The phytoene desaturase (*PDS*) gene, which encodes a key enzyme involving carotenoid biosynthesis, is commonly selected as the target gene for CRISPR/Cas9 experiments in plant, because the albino phenotype caused by the disruption of *PDS* can be easily recognized^16^. Previously, we cloned and analyzed two homologous gene copies in Chinese kale, namely *BaPDS1* and *BaPDS2*^33^. In this study, we utilized these two *BaPDSs* as target genes in *Agrobacterium*-mediated genetic transformation. The present study showed the simultaneous site-directed mutagenesis of these homologous genes in Chinese kale, which provided a theoretical foundation for the application of the CRISPR/Cas9 gene editing technique in Chinese kale and other *Brassica* vegetables.

## Results

### SgRNA design and vector construction

**Figure 1:**
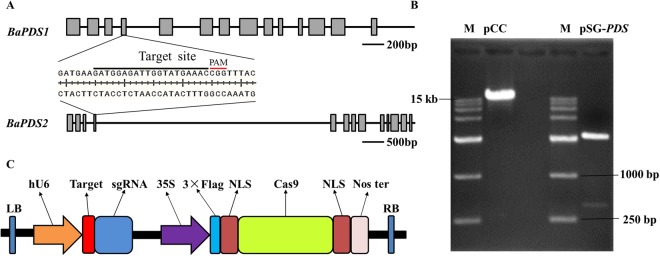
A map of the constructed vector. (**A**) Schematic of the *BaPDSs* gene fragment indicating the sgRNA target site and sequence.  indicates exon.  indicates intron. (**B**) Gel electrophoresis of the recombinant plasmid of pSG-*BaPDS* and pCC. M, DL15000 marker. (**C**) Structure of the CRISPR/Cas9 binary vectors for Chinese kale transformation. The Cas9 cassette was driven by the 35S promoter, while sgRNA was controlled by the hU6 promoter. The 3 × Flag is a label which can be used to identify the transgenic plants. NLS, nuclear localization sequence.

The common target site was selected in the fourth exon sequence of both *BaPDS1* and *BaPDS2*. The target sequence is GATGGAGATTGGTATGAAAC**CGG** (Fig. 1A). The target sequences for *BaPDS1* and *BaPDS2* are completely identical, and the GC content of gRNA is 40%. The recombinant plasmids of pSG-*BaPDS* and pCC were digested with *Eco*RI-HF and *Xba*I-HF, respectively (Fig. 1B). The fragment of the sgRNA expression cassette was purified by gel recovery and ligated into the digested pCC. The recombinant plasmid of pCC-target-sgRNA was successfully obtained for subsequent analysis (Fig. 1C).

### CRISPR/Cas9 transformation efficiency in Chinese kale

**Figure 2:**
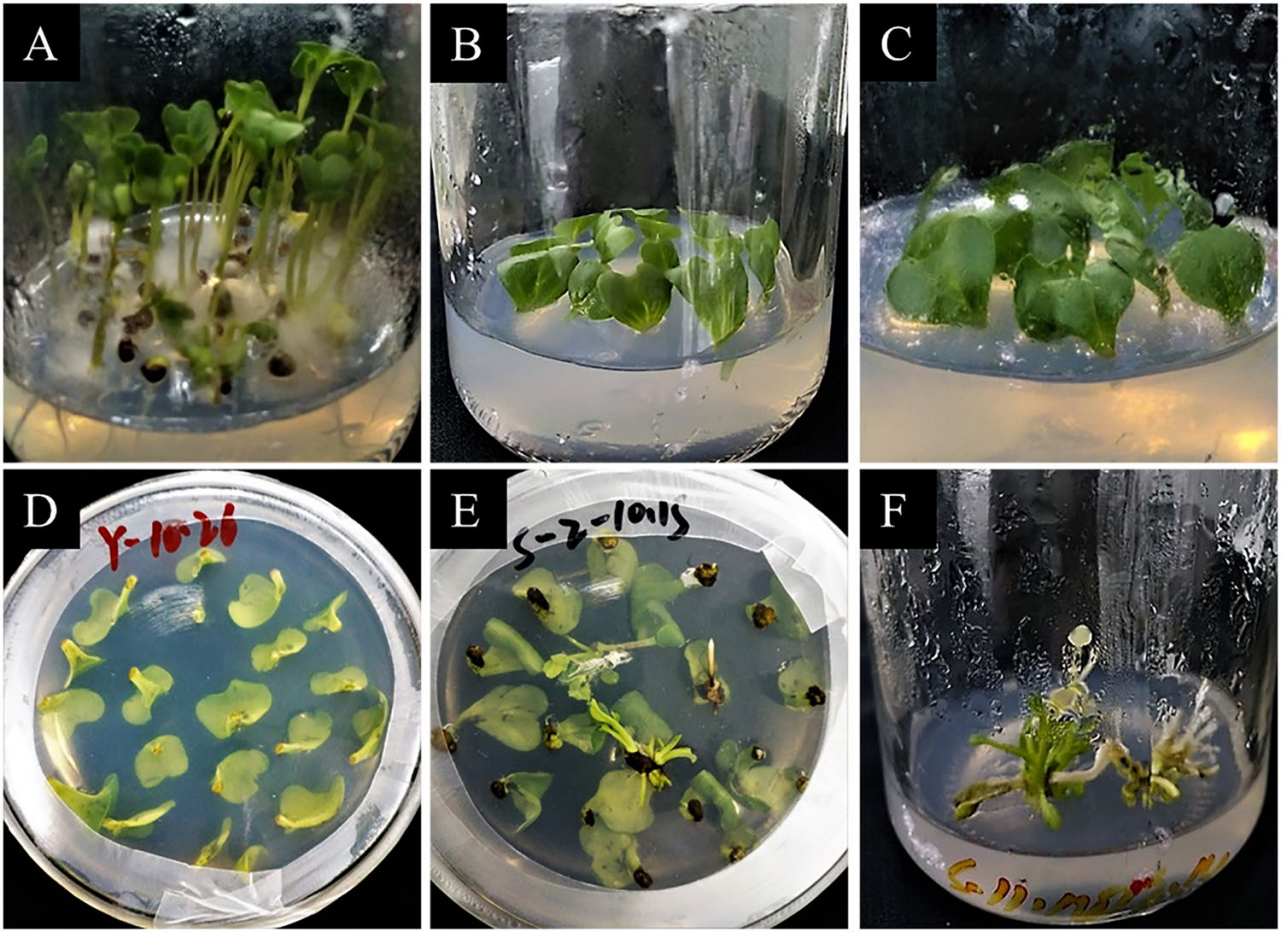
The procedure of *Agrobacterium*-mediated genetic transformation in Chinese kale. (**A**) Aseptic seedling culture, (**B**) pre-culture, (**C**) co-culture, (**D**) delayed screening, (**E**) resistance screening, (**F**) subculture.

**Figure 3:**
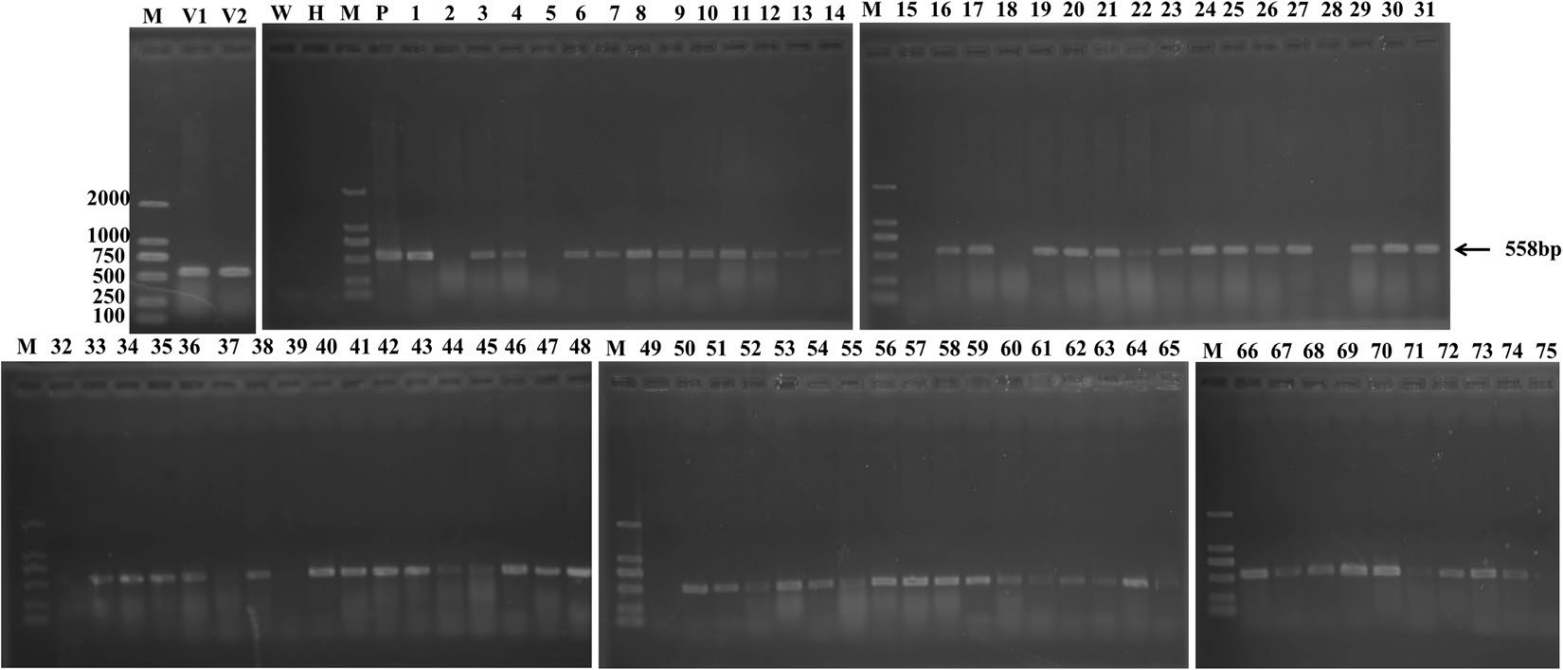
PCR detection of the hygromycin-resistant gene for the estimation of transformation efficiency. M: DL2000 maker; V1, V2: empty vector was used as a positive control; P: plasmid containing the vector and sgRNA also was used as a positive control; H,W: H_2_O and the gDNA of the wild-type was used as a negative control; 1–75: indicates the resistant plant line number. The arrow points to the aim band.

Using the established genetic transformation protocol of our laboratory (Fig. 2), about 5,000 explants were used for *Agrobacterium*-mediated transformation. Seventy-five hygromycin-resistant plants were successfully obtained (1.5%). To confirm the existence of the transformed constructs in the transgenic lines, genomic DNA was extracted from each hygromycin-resistant plant. PCR amplification with specific primers targeting the hygromycin gene was conducted. A 558-bp target band was successfully amplified using the empty vector, plasmid (positive control), and the 68 resistant plants. No products were obtained with the template in water, wild-type plants (negative control), and seven out of the 75 plants (lines 2, 5, 15, 18, 28, 32, and 49) (Fig. 3, Supplemental Fig. 1). These results indicated that the target expression cassette was successfully transferred into the 68 Chinese kale lines. In addition, the false positive rate in this experiment was only 9.33%.

### Statistical analysis of the *BaPDSs* mutation efficiency

To detect the mutation efficiency of CRISPR/Cas9, PCR was performed using specific primers (Table 1) and the 68 transgenic plants, followed by sequencing of the resulting PCR products. When both genes were simultaneously mutated, they were called double mutations, and when there was only one gene mutated, they were called single mutations. Approximately 76.47% of the transgenic plants harbored mutations, including 14 that were *BaPDS1* and *BaPDS2* double mutants, accounting for 20.59% of the total number of transgenic plants; 10 *BaPDS1* single mutants, accounting for 14.70%; and 28 *BaPDS2* single mutants, accounting for 41.18% (Table 2). Overall, the ratio of double mutations accounted for about one-fifth of the transgenic plants, and the proportion of the single mutant-type was more than a half, indicating that CRISPR/Cas9 system-induced gene editing has superior mutation efficiency in Chinese kale.

**Table 1:** Primers used in the study.

Primer names	Sequence of primers (5′-3′)	Aims
sgRNA-*BaPDSs*-F	GATGGAGATTGGTATGAAAC	Synthesis of the target site
sgRNA-*BaPDSs*-R	GTTTCATACCAATCTCCATC	
*BaPDS1*-CRISPR test-F	CCTGCAAAGCCTTTAAAAGTTGTCATT	Detection of the mutation in transgenic plants
*BaPDS1*-CRISPR test-R	CCAAGTTCTCCAAATAAGTTCTGCACG	
*BaPDS2*-CRISPR test-F	CCTGCAAAGCCTTTAAAAGTTGTGATC	
*BaPDS2*-CRISPR test-R	GCTATAGAAGATAAGAGCCGAGCCT	
Hyg-F	CGATTGCGTCGCATCGACC	Detection of the hygromycin resistance gene
Hyg-R	TTCTACAACCGGTCGCGGAG	
Off-target1-F	ATTCCTTGGAATTAGCTCTTCACTTGAC	Off-target analysis
Off-target1-R	GATGGGGACTCGAATCTTATCTGCC	
Off-target2-F	CAGGAATGCATGGGAAAGCATGAATG	
Off-target2-R	ATGATGCAACCGGGTAGTTTAATCG	
Off-target3-F	CATCATGAGTGGGGACAAGTTGTG	
Off-target3-R	TCAGCGTCCATGAGAGGTAAAGGG

**Table 2:** Percentage of transgenic plants examined with *BaPDSs* mutations in Chinese kale.

Mutation gene	Number of mutants	Mutation rate (%)	Total number of strains detected	Total mutation rate (%)
*BaPDS1* + *BaPDS2*	14	20.59	68	76.47
*BaPDS1*	10	14.70		
*BaPDS2*	28	41.18		

### Analysis of the *BaPDSs* mutations

**Figure 4:**
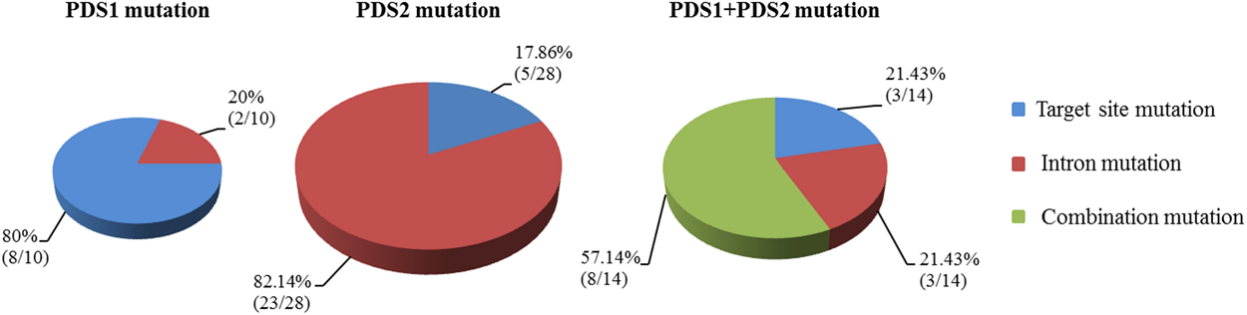
Analysis of the number and proportion of mutation sites in Chinese kale mutants. Mixed type means that one of the two genes is mutated at the target site and the other is mutated within an intron.

Fifty-two *BaPDSs* mutants were obtained by sequencing confirmation. The distribution of mutations of CRISPR/Cas9-editing of the two homologous gene copies was investigated (Fig. 4). Among the *BaPDS1* and *BaPDS2* double mutants, the proportion of both mutations at the target site or within introns respectively accounted for 21.43% (3/14) of the total number of mutations, and the proportion of mixed mutations was 57.14% (8/14). Among the *BaPDS1* single mutants, 80% (8/10) were situated at the target site, whereas 20% (2/10) occurred in intronic area. Among the *BaPDS2* single mutants, the frequency of mutations at the target site and within introns was 17.86% (5/28) and 82.14% (23/28), respectively (Fig. 4). These results suggest that the CRISPR/Cas9 system can simultaneously edit both copies of the genes using the sgRNA, which targets the consensus sequence of the two homologous genes in Chinese kale.

### Analysis of the *BaPDS1* mutations

**Figure 5:**
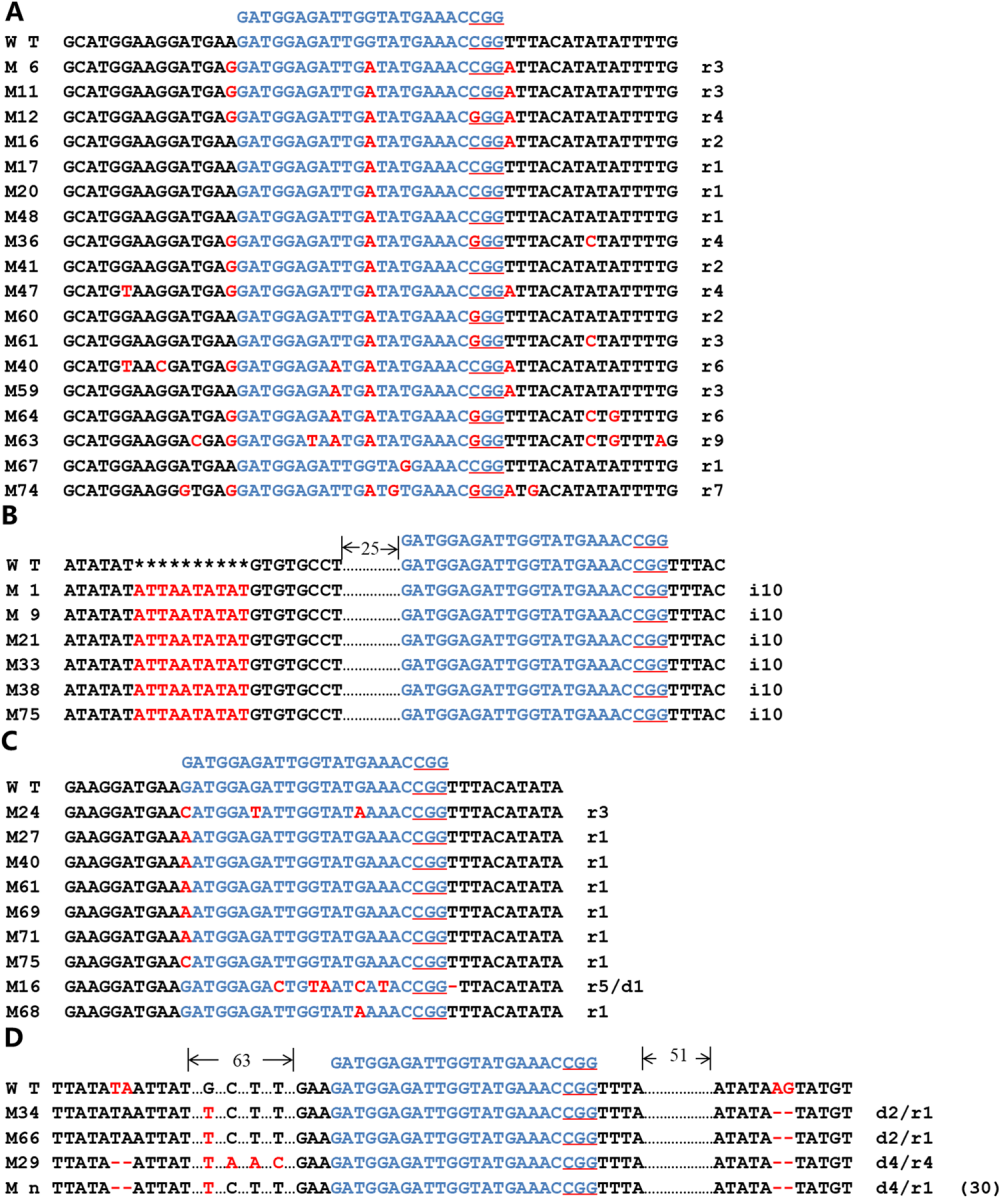
CRISPR/Cas9 system-induced mutation detection in Chinese kale mutants. (**A**) *BaPDS1* mutations at the target site; (**B**) *BaPDS1* mutations within introns; (**C**) *BaPDS2* mutations at the target site; (**D**) *BaPDS2* mutations within introns. The target sequence is indicated in blue, the PAM sequence (NGG) is underlined in red, mutated bases are indicated in red font, the short line represents the deletion base, and the asterisks indicate the spacing between bases. i #, # number of base insertions. r #, # number of base replacements. d #, # number of base deletions. The number of identical mutations is indicated in parentheses. Mn contains strains M4, M7, M8, M9, M10, M11, M12, M13, M20, M23, M26, M31, M32, M33, M36, M37, M38, M41, M42, M44, M45, M48, M50, M55, M57, M60, M65, M70, M72, M73.

**Figure 6:**
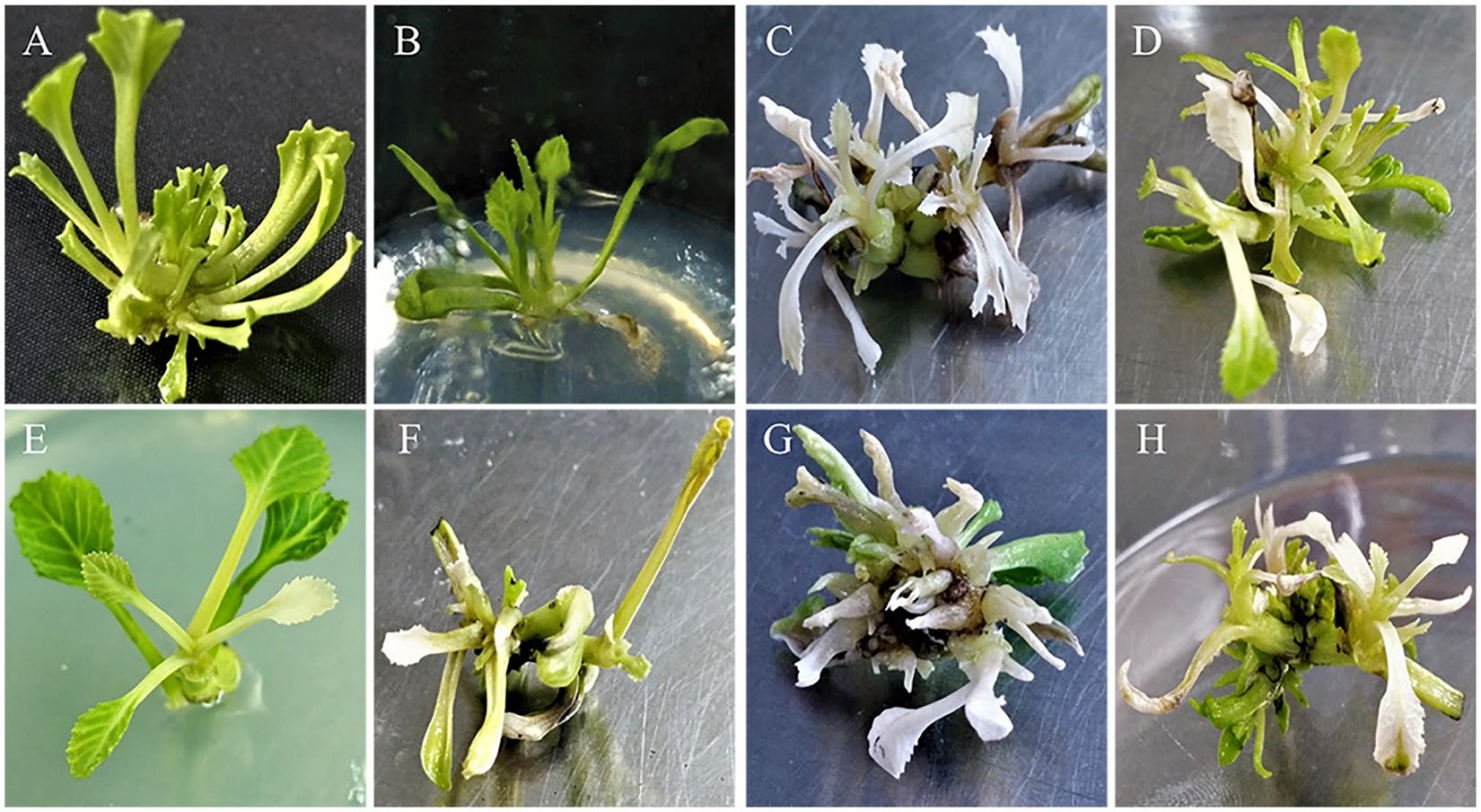
The albino phenotype of the *BaPDS* mutants after transformation with the CRISPR/Cas9 system. (**A**) Non-transgenic plant; (**B**) Transgenic plants with an empty vector (containing T-DNA); (**C**) *BaPDS1* and *BaPDS2* double mutant at the target site; (**D**) *BaPDS1* single mutant at the target site; (**E**) *BaPDS2* single mutant at the target site; (**F**) *BaPDS1* and *BaPDS2* double mutants within the introns; (**G**) *BaPDS1* single mutant within the introns; (**H**) *BaPDS2* single mutant within the introns.

The mode of the *BaPDS1* mutation was further analyzed. A total of 24 *BaPDS1* mutations were identified, including 18 that have one- to nine-base single-nucleotide substitutions at the target site (Fig. 5A). Interestingly, 17 mutants showed a G to A substitution at the ninth base upstream of the PAM locus. This substitution is predicted to result in an amino acid change from tryptophan (Trp) to a stop codon, thereby generating a truncated (nonsense) BaPDS1 protein. A single mutant harbored a G to T substitution six bases upstream of the PAM locus. Similarly, the encoded tyrosine (Tyr) was substituted by a stop codon, thereby also resulting in a truncated protein (Fig. 5A). In addition, we found that among the remaining six *BaPDS1* mutants, a 10-bp (ATTAATATAT) insertion was detected in the third intron of *BaPDS1* (Fig. 5B). However, regardless of the position of the mutation (target site or intron), the plants showed the same albino phenotype (Fig. 6D,G).

### Analysis of the *BaPDS2* mutations

Similarly, the mode of the *BaPDS2* mutations was also investigated. Among the 42 *BaPDS2* mutants, nine were found to be mutated at the target site, resulting in a frameshift mutation or amino acid substitution (Fig. 5C). Thirty-one mutants had an AG deletion in the fourth intron and a TA deletion in the third intron, and the remaining two had an AG deletion in the fourth intron. (Fig. 5D). Similarly, regardless of the site of the mutation, *BaPDS2* single mutants exhibited a similar albino phenotype (Fig. 6E,H).

### Patterns and frequency of the *BaPDSs* mutations

**Figure 7:**
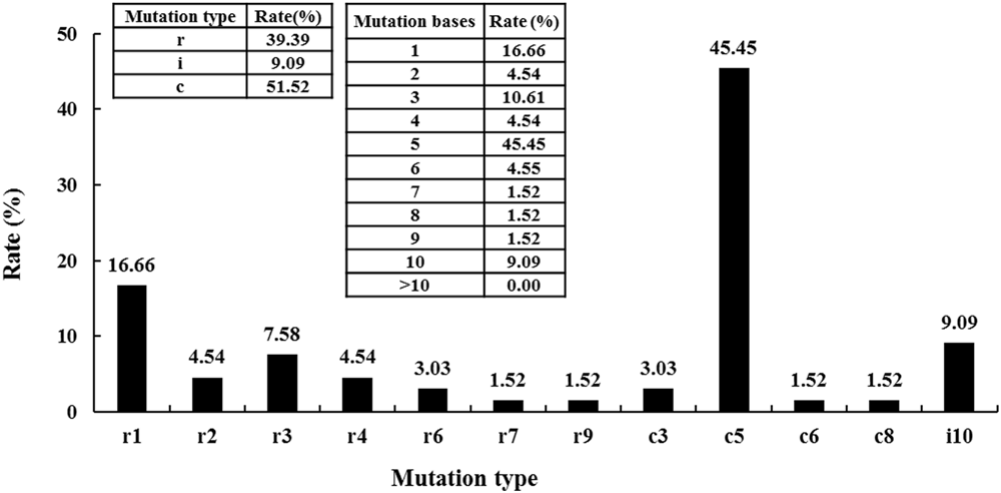
Types and frequency of CRISPR/Cas9-mediated mutations in Chinese kale. The graph includes the sequencing data of all of the mutants. The illustration on the left shows the types of mutations. The illustration on the right shows the frequency of the mutations of different lengths. *X*-axis: r #, the number of base replacements; c #, the base number of combined mutations; i #, the number of base insertions.

A variety of mutation types were observed in this survey, including substitutions, insertions, and combinatorial mutagenesis (substitutions and deletions occurring simultaneously) (Fig. 7). Among these, up to 51.52% of the mutations were combined mutations, followed by substitutions (39.39%). Insertion mutations accounted for only 9.09% of the observed genomic alterations. All of the CRISPR/Cas9-mediated mutations were short nucleotide changes (≤10 bp) (100%), and 5-bp-long combined mutations were the predominant type, accounting for 45.45% of the observed mutations. All of the insertion mutations were involved in the ATTAATATAT sequence.

### Analysis of potential off-targets

To assess whether the CRISPR/Cas9 system specifically edits the *BaPDS* genes, three potential off-target sequences were selected using the CRISPR-P web tool. Reports have indicated that the ‘seed sequence’, which consists of 12 nucleotides located at the target site and adjoining the PAM, is crucial for specific recognition and efficient targeted cleavage of the Cas9 protein^15,34^. Among the three potential off-target sites selected, off-target sites 1 and 3 only showed a 1-bp mismatch in the seed sequence, and off-target site 2 had a 2-bp mismatch in the seed sequence (Table 3). All three potential off-target sites were PCR amplified using specific primers (Table 1). Sequencing did not detect any mutations within all of the tested sites. These results indicate that CRISPR/Cas9-induced target mutagenesis is highly specific to the *BaPDSs* in Chinese kale and does not have off-target effects.

**Table 3:** Potential off-target analysis.

Target	Sequence	Mismatches	Efficiency
Target site	GATGGAGATTGGTATGAAACCGG		
Off-target site1	**T**AT**A**G**T**GATT**A**GTATGAAACGGG	4	0.0%
Off-target site2	**A**A**A**GGAGATTG**CA**ATGAAACTGG	4	0.0%
Off-target site3	G**G**TGGAG**C**TTG**T**TATGAAACTGG	3	0.0%

The seed sequence is underlined.

### Albino phenotype of the *BaPDSs* mutants

All of the obtained 52 *PDS* mutants showed a clear albino phenotype compared to the wild-type (Fig. 6). Interestingly, the leaves of plants with edited *PDS1* and *PDS2* were pure albino (Fig. 6C), whereas those with *PDS1* or *PDS2* single mutations either at the target loci or within introns resulting in a mosaic albino phenotype belonged to chimeric mutants (Fig. 6D–H). These results indicate that the gene function of *BaPDSs* had been knocked-out or knocked-down.

## Discussion

*Brassica* vegetables have drawn increasing attention owing to its high economic value. Based on morphological characteristics, they are divided into three types of *B. rapa*, *B. oleracea*, and *B. juncea*. Chinese kale is one of the most important vegetables within *B. oleracea*^31,32^. It has 18 chromosomes and belongs to the CC genome type^35^. As a diploid, Chinese kale is suitable for gene modification and offspring inheritance studies. The screening of targeted mutants has long been employed as a useful strategy for plant functional genomics and crop improvement. However, this approach is extremely laborious and time-consuming as it generally entails traditional mutagenesis methods^36,37^. In recent years, precise, efficient, and multi-site mutagenesis has been achieved in plants using genome editing technology. As the third-generation genome editing technology, the CRISPR/Cas9 system has been applied in various plants since its emergence. Numerous studies have used protoplast transient transformation to integrate the CRISPR/Cas9 system, which can rapidly and efficiently screen the target site and detect mutations^13,18^. However, unlike model plants such as *Arabidopsis* and tobacco, protoplast technology (isolation, purification, and especially regeneration) remains a major obstacle in accessing mutated *Brassica* vegetables, thereby limiting its practical application to this particular crop species. Therefore, *Agrobacterium*-mediated stable transformation was selected in this study, despite the efficiency of transient transformation being higher than that of stable transformation^38^. As expected, a variety of Chinese kale mutants were obtained using the CRISPR/Cas9 system, with relatively high efficiency (76.47%).

Multi-site precise editing is very important for investigations into complex traits and functionally redundant genes. However, the high genetic redundancy in *Brassica* crops makes it difficult to alter a trait by random mutagenesis and to obtain corresponding mutants, especially recessive mutants^28,39^. The innate ability of Cas9 to simultaneously edit multiple loci within the same individual has numerous potential applications, such as the mutation of multiple members of a gene family or functionally related genes that control complex traits^40^. Targeted mutagenesis of duplicated genes in *Camelina sativa* was conducted using CRISPR/Cas9 gene editing to generate triple mutants of the three delta-12-desaturase (*FAD2*) genes^39^. A four-allele mutated line of the granule-bound starch synthase (*GBSS*) gene obtained by CRISPR/Cas9 resulted in the complete knockout of GBSS enzyme activity in potato^14^. In this study, double mutants of *BaPDS1* and *BaPDS2* were obtained, and their proportion amounted to about 20% of the total number of transgenic plants (Table 2). In this study, we selected only one common and conservative co-target site in two homologous genes (*BaPDS1* and *BaPDS2*) of Chinese kale rather than using the tandem multiplex gRNA method that has been employed in previous studies^13,41^ because the mutation efficiency induced by multiplex gRNA is significantly lower than that of a single gRNA^13^. Our results confirmed the powerful capability of the CRISPR/Cas9 system in inducing multiplex mutagenesis simultaneously, and furtherly broadened its application in various plant species.

Mutation types are affected by different factors and vary among plant species. Previous reports have shown that 1-bp insertions and deletions predominantly occur in rice^11^ and *Arabidopsis*^17^, deletions involving a few bases are the major types of mutations in tobacco^13^, and the proportion of substitutions induced by *BnaA6.RGA-sgRNA1* in *B. napus* are much higher than other sgRNAs^42^. In this study, short nucleotide substitutions predominantly occurred in Chinese kale. This discrepancy may be caused by differences in intrinsic DNA-repair mechanisms among plant species, transformation methods, target genes, or target sites^11,42^.

In this study, some mutations did not occur precisely at the target site, but within flanking regions. The *BaPDS2* intronic mutation in 33 lines involved the sequence AAG (Fig. 5D), which is another PAM (NAG)^43^ besides NGG^44^ for Cas9 cleavage. Conversely, there were six lines that harbored a 10-bp insertion (ATTAATATAT). The mutation involved the third intron of *BaPDS1*, which is AT-rich (Fig. 5B). Furthermore, there were 31 *BaPDS2* mutations with a 2-bp deletion (TA) in the third intron, which is also AT-rich (Fig. 5D). Most of these mutations occurred at AT-rich sites. These findings indicate that Cas9 preferentially cuts and edits AT-rich sequences in Chinese kale, which is similar to that observed in rice^11^, citrus^45^, and *Camelina*^46^. However, this hypothesis requires further confirmation.

Off-target effects are usually caused by the base-mismatches between sgRNA and the genomic DNA sequences, which hinders further application of the CRISPR/Cas9 technology^47,48^. However, there is growing evidence that off-targets might not be a critical problem in plants because its actual risk may be low during tissue culture-based transformation or other mutagenesis treatments^40^. In this study, we also assessed for potential off-target sites, but found none. These results corroborate the previous conclusion that Cas9/sgRNA specificity is determined by the position and number of mismatches in sgRNA target pairing^15^, and a well-designed specific sgRNA, such as the sgRNA in this study, would not target non-targeted sites^11^. However, this study was not a comprehensive evaluation of the specificity for targeted gene editing using the CRISPR/Cas9 system in Chinese kale, and more candidate off-target sites need to be analyzed in the future.

Numerous studies have shown that intronic sequences are closely related to the expression of plant functional genes. Recent investigations also have revealed that a large number of microRNAs are located within the introns of protein-coding genes, linking their expression to the promoter-driven regulation of the host gene^49,50^. For instance, miR838 that is derived from a hairpin within intron 14 of the *DC1* could mediate gene expression by auto-regulation in *A. thaliana*^51^. In this study, we found several mutations within the third and fourth introns of *BaPDSs*, and these mutation modalities were relatively higher in frequency (Fig. 5B,D). Furthermore, all of the intron mutations resulted in mosaic albino phenotypes (Fig. 6F–H), indicating a knock-down in the function of the *BaPDS* genes. These introns may be involved in gene expression in Chinese kale. Nevertheless, the reasons why intron mutations cause loss of gene function remain unclear and will be further studied in subsequent studies.

Generating distinct mutants of multiple copies of homologous genes could be helpful in elucidating the function of the respective genes, and successful examples in plants, such as *Camelina sativa*^39^ and potato^14^, were previously reported. Although we have cloned *BaPDS1* and *BaPDS2* in Chinese kale, as well as analyzed their expression patterns in an earlier study^33^, their respective functions remain elusive. As expected, all three types of mutants (*PDS1* and *PDS2* double mutations and *PDS1* or *PDS2* single mutations) were obtained simultaneously in this study. The pure albino phenotype of the double mutants and the mosaic albino phenotype of the *BaPDS1* and *BaPDS2* single mutants (Fig. 6) indicate that the functions of *BaPDS1* and *BaPDS2* are distinct and also partially compensated. In addition, the loss of function of either gene would be lethal to Chinese kale plants, suggesting that both are essential for carotenoid biosynthesis.

The rapid development of the CRISPR/Cas9 system has provided a robust platform for plant genomic studies. The present investigation achieved fixed-point editing of the Chinese kale genome using the CRISPR/Cas9 system via stable transformation for the first time. Additionally, a single target site for simultaneously mutating multi-genes to create various mutation materials was also achieved. The findings of the present study strengthen the application of the CRISPR/Cas9 system in cultivated *Brassica* vegetables, and enrich our understanding of the mutation mode of CRISPR/Cas9 in *Brassica* crops, and provide a foundation for the further study of gene function through the creation of mutants.

## Materials and Methods

### Plant materials

The Chinese kale cultivar ‘Sijicutiao’ which is a multi-generation inbred from our laboratory was used in the study. Sterile seedlings grown on 1/2 Murashige and Skoog (1/2 MS) medium with 0.8% phytagar, and they were placed on tissue culture chambers with temperature of 25/20 °C (day/night), light cycle of 16/8 h (day/night), and light intensity of 100 μmol m^−2^ s^−1^. After seven days, cotyledons with 1- to 2-mm long petioles were cut and used as explants in *Agrobacterium*-mediated transformation.

### Vector construction

The CRISPR/Cas9 vector supports from our laboratory^52^. The plasmid pX330, pCAMBIA1302, and pUC19 were used as materials for the construction of the CRISPR/Cas9 vector using recombinant DNA technology. The mgfp sequence in pCAMBIA1302 was successfully replaced by the Cas9 sequence from pX330, and the recombinant plasmid was constructed, hereafter referred to as pCC. The sgRNA sequence in pX330 was imported into the multiple cloning site region of the pUC19 plasmid, and the recombinant plasmid was designated as pSG. pCC and pSG comprised the two basic vectors of the CRISPR/Cas9.

According to our previous report, the *BaPDS* gene family has two members, *BaPDS1* (GenBank Accession No. KX426039) and *BaPDS2* (GenBank Accession No. KX426040)^33^. Sequence analysis of *BaPDSs* was conducted using the web tools of CRISPR DESIGN (http://crispr.mit.edu/) and ZiFiT Targeter Version 4.2 (http://zifit.partners.org/ZiFiT/CSquare9 Nuclease.aspx), and the common target sequence of *BaPDS1* and *BaPDS2* was designed. The synthesized oligos were annealed and inserted into the *Bbs*I sites of the pSG. Then, the recombinant plasmid (pSG-*BaPDS*) was digested by *Eco*RI-HF and *Xba*I-HF, and the sgRNA expression cassette was inserted into the multiple cloning site region of the pCC vector to generate pCC-target-sgRNA for Chinese kale transformation. All of the above vectors were verified by restriction and sequencing.

### *Agrobacterium*-mediated transformation of Chinese kale plants

The transformation of Chinese kale was conducted as previously described, with modifications^53,54^. The explants pre-cultured for 3 d in Murashige and Skoog (MS) medium with 0.5 mg/L 2,4-D and 0.8% phytagar were infected with the *Agrobacterium* strain GV3101 (optical density at 600 nm between 0.6 and 0.8) by immersion for 1–2 min. The explants were co-cultivated with *Agrobacterium* strain GV3101 for three days in MS medium with 0.03 mg/L naphthaleneacetic acid (NAA), 0.75 mg/L boric acid (BA), and 0.8% phytagar, and then transferred to the MS medium supplemented with 0.03 mg/L NAA, 0.75 mg/L BA, 0.8% phytagar, 325 mg/L carbenicillin, and 325 mg/L timentin for a week. Hygromycin-resistant shoots regenerated on the same medium with 12 mg/L hygromycin B were transferred to tissue culture bottles containing the subculture medium. After three months, hygromycin-resistant plantlets were obtained and prepared for subsequent analysis.

### Transformation efficiency detection

Genomic DNA was extracted from the shoots of hygromycin-resistant and wild-type plants using a standard cetyltrimethylammonium bromide (CTAB) method. To confirm the Chinese kale transformants, PCR amplification using the specific primers HygR-F and HygR-R (Table 1), which were designed to flank the hygromycin sequence contained in the vector, was performed. The PCR products were detected using electrophoresis to calculate transformation efficiency. The transformants without the hygromycin gene were not used for further analysis.

### Mutation detection

To evaluate mutations introduced in the CRISPR/Cas9 transgenic plants, genomic DNA of each positively transgenic shoot was amplified using specific primers, which were designed to amplify the 816-bp and 702-bp flanking regions of the target site of *BaPDS1* and *BaPDS2*, respectively. For genotyping of the transgenic plants, the PCR products were sequenced. DNAMAN software (version 6.0; Lynnon Corporation, Canada) was used to compare the sequence, the mutation rate was calculated, and all of the sequencing data were collected to analyze the mutation type.

### Off-target analysis

Potential off-target sites were identified with the CRISPR-P^55^ online software (http://crispr.hzau.edu.cn/CRISPR2/) using the full 20-bp target sequence in a BLAST analysis. These top-ranking potential off-target sites containing 1–2 bp mismatches in the 12-bp seed sequence were selected (Table 3). The sequences surrounding the potential off-target sites were amplified using specific primers (Table 1), and the PCR products were analyzed by Sanger sequencing.

## Electronic supplementary material

Figure S1. Original image shows the PCR detection of the hygromycin-resistant gene for the estimation of transformation efficiency.
