# Supplementary material for: CRISPR/Cas9-mediated mutagenesis of homologous genes in Chinese kale
Source: Sci Rep. 2018 Nov 14;8:16786. doi: 10.1038/s41598-018-34884-9 (PMC6235979; doi:10.1038/s41598-018-34884-9)
Supplement: Supplementary file 1 — Figure S1. Original image shows the PCR detection of the hygromycin-resistant gene for the estimation of transformation efficiency. [file 41598_2018_34884_MOESM1_ESM.pdf]

Title page

CRISPR/Cas9-mediated mutagenesis of homologous genes in Chinese kale

Bo Sun[1,†], Aihong Zheng[1,†], Min Jiang[1], Shengling Xue[1], Qiao Yuan[1], Leiyu Jiang[1], Qing Chen[1],

Mengyao Li[1], Yan Wang[2], Yong Zhang[1], Ya Luo[1], Xiaorong Wang[2], Fen Zhang[1,*], and Haoru Tang[1,2,*]

[1]College of Horticulture, Sichuan Agricultural University, Chengdu 611130, China

[2]Institute of Pomology and Olericulture, Sichuan Agricultural University, Chengdu 611130, China

[†] These authors contributed equally.

Supplemental Information

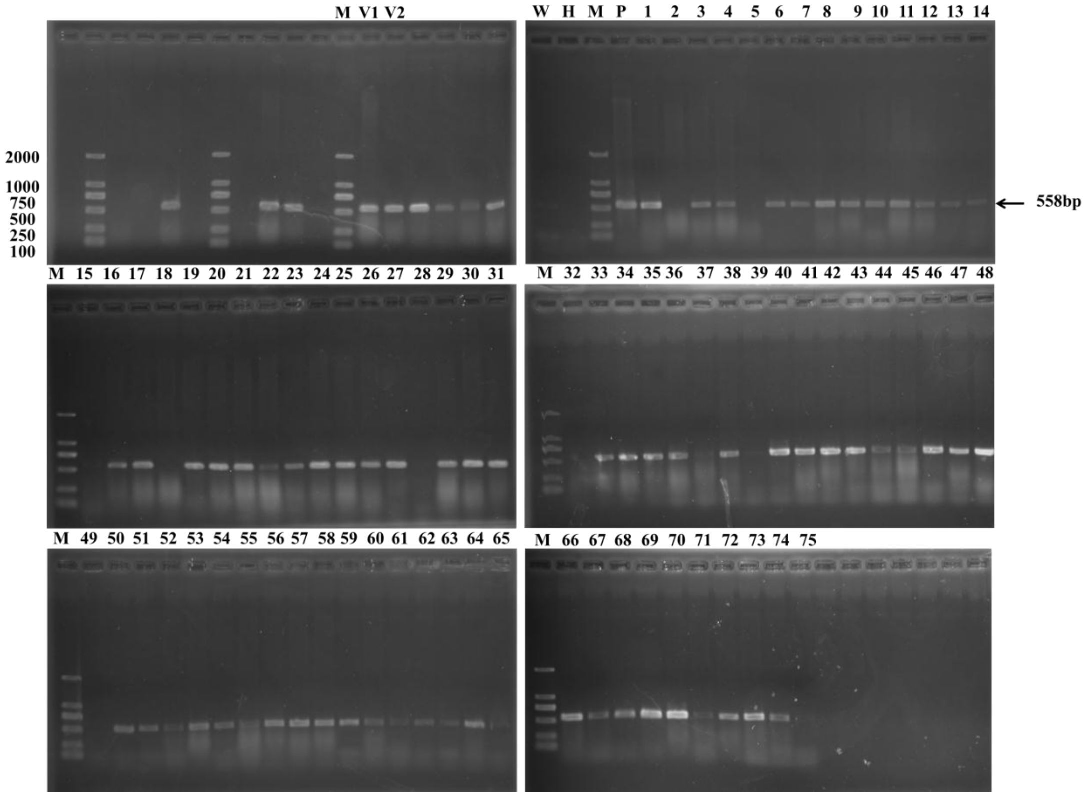

Figure S1. Original image shows the PCR detection of the hygromycin-resistant gene for the estimation of transformation efficiency.

M: DL2000 maker; V1,V2: empty vector was used as a positive control; P: plasmid which contain vetor and sgRNA also was used as a positive control; W: gDNA of WT was as a negative control; H: H_2O was as a negative control; 1-75: indicates the resistant plant line number; The arrow points to the aim band.
